# Melanocortin-4 receptor (MC4R) rs17782313 polymorphism interacts with Dietary Approach to Stop Hypertension (DASH) and Mediterranean Dietary Score (MDS) to affect hypothalamic hormones and cardio-metabolic risk factors among obese individuals

**DOI:** 10.1186/s12263-020-00672-2

**Published:** 2020-08-05

**Authors:** Mahdieh Khodarahmi, Mohammad Asghari Jafarabadi, Mahdieh Abbasalizad Farhangi

**Affiliations:** 1 Nutrition Research Center, Department of Nutrition, Faculty of Nutrition and Food Science, Tabriz University of Medical Sciences, Tabriz, Iran; 2 Road Traffic Injury Research Center, Tabriz University of Medical Sciences, Tabriz, Iran; 3 Department of Statistics and Epidemiology, Faculty of Health, Tabriz University of Medical Sciences, Tabriz, Iran; 4 Drug Applied Research Center, Tabriz University of Medical Sciences, Attar-neishabouri Ave, Golgasht St, Tabriz, 5165665931 Iran

**Keywords:** Diet quality, Obesity, Gene-diet interaction, MC4R, Cardio-metabolic risk

## Abstract

**Background and aim:**

The association with obesity of a common variant near the melanocortin-4 receptor (MC4R) gene (rs17782313) has been indicated in various studies. Adherence to dietary quality indices also have shown to have potential favorable effects on obesity-related health outcomes. However, no study has examined the interaction between rs17782313 and the Dietary Approach to Stop Hypertension (DASH) score and the Mediterranean Dietary Score (MDS) on cardio-metabolic risk factors and hypothalamic hormones. Therefore, the purpose of the current study was to examine whether adherence to these dietary quality indices modifies the association of the MC4R rs17782313 polymorphism with cardio-metabolic risk factors and hypothalamic hormones among obese adults.

**Method:**

Two hundred eighty-eight healthy obese adults were recruited in this cross-sectional study. Diet quality indices, including DASH score and MDS, were calculated from a validated 147-item food frequency questionnaire (FFQ). MC4R s17782313 genotypes were determined by polymerase chain reaction-restriction fragment length polymorphism (PCR-RFLP). An ANCOVA multivariate interaction model was used to assess the gene-diet interaction.

**Results:**

Significant interactions were detected between DASH score and MC4R rs17782313 genotypes on systolic blood pressure (SBP), atherogenic index of plasma (AIP), and serum glucose and triglyceride (TG) among the female group (*p*_Interaction_ < 0.05). In the male group, there were gene-DASH and gene-MDS interactions in relation to serum glucose concentration and plasma α-melanocyte stimulating hormone (MSH) levels, but these were found only in multi-adjusted interaction models (*p*_Interaction_ < 0.05). In addition, there was a significant interaction between MC4R rs17782313 polymorphism and DASH score on plasma agouti-related peptide (AgRP) concentrations in the female group in a multivariate interaction model (*p*_Interaction_ < 0.05). An inverse association between DASH score and chance of having the CC genotype in a multivariate-adjusted model among women was also revealed.

**Conclusion:**

MC4R rs17782313 interacts with healthy dietary pattern (DASH score and MDS) to influence cardio-metabolic risk factors and hypothalamic hormones in obese individuals. Prospective cohort studies are needed to further assess these findings.

## Introduction

Obesity is a worldwide pandemic, and its rates have increased rapidly in recent years among all age groups [1]. The prevalence of obesity is increasing in Iran as in other developing nations and is estimated to affect over 21.7% of adult Iranians [2]. Obesity is a complex disorder and is known to be a major cause of cardio-metabolic risk factors such as hypertension, insulin resistance, and hyperlipidemia, and hence it is accompanied with increased risk of non-communicable diseases (NCD) such as cardio-vascular disease (CVD), type 2 diabetes, and metabolic syndrome [1].

Obesity is a multifactorial disorder believed to be determined both by genetic and environmental factors and their complex interactions [3]. Manifestation of the obesity phenotype in susceptible subjects depends in part on environmentally modifiable determinants such as diet [4, 5]. One of the strongest genes increasing susceptibility to obesity is the melanocortin-4 receptor (MC4R). According to a large meta-analysis of genome-wide association studies (GWAS), a common genetic variant near MC4R (rs17782313) was found as a second association signal following FTO rs9939609 for common obesity [6]. MC4R, a plasma membrane G protein-coupled receptor that is expressed widely in endocrine regions of the brain, plays a main role in regulation of food intake and energy expenditure [7]. MC4R, as a part of the leptin-melanocortin signaling system, is modulated by endogenous antagonist agouti-related protein (AGRP) and agonist α-melanocyte stimulating hormone (α-MSH) actions [8]. The binding of α-MSH to MC4R by acting as anorexigenic neuropeptide leads to the inhibition of energy intake and an increase in energy expenditure [9]. On the other hand, AgRP acts as orexigenic neuropeptide and leads to increased appetite and suppressed metabolic rate [9]. There is growing evidence that rs17782313, a variant mapped 188 kb downstream of MC4R, is related to high dietary intake [10] and different obesity-related phenotypic traits [11]. Subsequent analyses have indicated that cardiovascular risk factors, such as insulin resistance [12], type 2 diabetes [13], and hypertriglyceridemia [14], are associated with risk allele C for MC4R rs17782313. Although these associations were successfully replicated among various ethnic populations [15], the results were inconclusive [16, 17]. These inconsistent results suggest that environmental factors, such as diet, may modulate the effects of MC4R gene polymorphisms and change the genetic susceptibility to obesity and related diseases. Likewise, understanding gene-diet interactions may aid in prevention of obesity-related phenotypes and their better management.

The role of diet as a lifestyle factor in preventing obesity and related non-communicable diseases as well as preserving health is indisputable [18]. Since nutrients, food, and food groups are not usually consumed in isolation, and also due to synergistic and interactive effects of many components of diet, studying dietary patterns, which account for complexity of diet, provides the opportunity to assess the real effects of diet on health outcome [19]. Among a priori-defined dietary pattern approaches, as known as “diet quality indices,” the Dietary Approach to Stop Hypertension (DASH) score and Mediterranean Dietary Score (MDS) have been known for being cardio-protective [20]. These indices are widely used in epidemiological research [21]. Research using the above indices has shown that high compliance with these healthy dietary patterns was related to a decrease in the risk of cardiovascular disease, cancer, and other major chronic diseases [22, 23]. Nevertheless, the results of studies regarding the association between the above-mentioned diet quality indices and obesity and other related diseases are not consistent [24, 25]. Additionally, studies using these health indices in Middle Eastern populations are scarce [26], and the majority of research in this area has been carried out in Western countries [23]. Evidence also exists, however, that general healthy dietary recommendations may not be useful for every person due to varying genetic structures [27]. All of the above-mentioned suggest the existence of gene-diet interactions for obesity and related cardio-metabolic traits. Hence, studying overall diet in the form of diet quality indices could lead to better identification of gene-diet interaction and to generate personalized dietary recommendations for the prevention of obesity and related chronic phenotypes. To the best of our knowledge, only a few studies have evaluated the interaction between the rs17782313 variant and nutrient intake on obesity and its related phenotypes [28]. Although numerous studies have been carried out in different populations [10–28], none of these studies have specifically evaluated interactions between adherence to diet quality indices and rs17782313 polymorphism on cardio-metabolic risk factors and hypothalamic hormones (α-MSH and AgRP). Therefore, the aims of the current study were (1) to evaluate the association between genotypes of rs17782313 and cardio-metabolic risk factors and diet quality indices (DASH score, MDS) and (2) to examine potential interactions between genotypes of rs17782313 and diet quality indices on cardio-metabolic risk factors and hypothalamic hormones in a group of obese Iranian subjects.

## Methods

### Study design and participants

For this cross-sectional study, 288 healthy individuals (51.1% males and 48.9% females) aged 20–50 were recruited using convenience sampling through announcements. These announcements contained primary information about inclusion criteria (age 20 to 50 years, good health, and obesity (BMI ≥ 30 kg/m2)) were placed in public places and health care facilities. The details of participant recruitment have been detailed elsewhere [5]. Exclusion criteria included a history of cardiovascular disease, hypertension, cancer, type 2 diabetes mellitus, renal disease, pregnancy and lactation, and any medications effective for weight loss such as loop diuretics, cortico-steroids, or antidepressants. Additionally, subjects who had any recent surgery such as bariatric were also excluded. The study protocol was approved by the Ethical Committee of the Tabriz University of Medical Sciences (registration code IR.TBZMED.REC.1396.768), and written informed consent was obtained from all participants prior to participation in the study.

### Dietary assessment and dietary score calculation

A trained doctoral-level nutrition student collected the information through face-to-face interviews. The customary dietary intake over the last year was evaluated using a reliable and validated 147-item semi-quantitative food frequency questionnaire (FFQ) [29, 30]. A trained dietitian asked the participants to select the frequency and amount of the intake of each food item consumed during the previous year on a daily, weekly, or monthly basis. Household measures were used to convert the portion sizes of reported foods to grams. Daily energy and nutrient intake were analyzed using the Iranian Food Composition Table (FCT) [31] and complemented with the US Department of Agriculture FCT [32].

### DASH score

The DASH score, a measure of adherence to the DASH diet, was constructed based on a method developed by Fung et al. [33]. This indicator was developed according to eight components (seven food groups and one nutrient) that are either emphasized or minimized in the DASH diet. This approach encourages intake of nuts and legumes, fruits, vegetables, low-fat dairy products, and whole grains, while it discourages intakes of red and processed meats, sweetened beverages, and sodium. For each of the above components, sex-specific quintile rankings were performed for all study participants. For the emphasized components, including fruits, vegetables, whole grains, low-fat dairy, and nuts and legumes, individuals in the highest quintile of intake were given a score of 5, and subjects with the lowest quintile received a score of 1. A reverse-scoring method was applied for the remaining components (red and processed meats, sweetened beverages, and sodium). A total DASH score was obtained by summing the scores of the eight dietary components, with a total possible score ranging from 8 to 40 points. A higher overall DASH score represents better diet quality and higher adherence to the DASH diet.

### MDS

MDS, a 9-point index proposed by Trichopoulou et al., was calculated to assess the degree of adherence to the Mediterranean diet [34]. The scoring system of this 9-point scale is based on sex-specific median intakes of its components (vegetables, legumes, fruits and nuts, cereals, fish, meats, dairy products, alcohol, and the ratio of monounsaturated fatty acid (MUFA) to saturated fatty acid (SFA)). In the present study, since there was no reliable data on alcohol consumption, the alcohol component was eliminated. The scoring system was modified for a total score of 8 points. In this index, a score of 1 was assigned to protective components (i.e., nuts and fruits, vegetables, legumes, cereals, fish, and MUFA/SFA) for intake equal to or above and a score of 0 for intake equal to or below the sex-specified median. Conversely, for non-protective components (like meats and dairy products), the scoring was reversed. Component scores were summed to obtain overall MDS, ranging from 0 (lowest conformity) to 8 (highest conformity).

### Demographic, anthropometric, and blood pressure measures

Demographic information such as age, gender, marital status, history of smoking, and medical history were collected using questionnaires by a trained interviewer. Additional data regarding socio-economic status (SES) was collected regarding occupational position, educational status, family size, and house ownership, which were considered as individual indicators. A total score for SES was then categorized into three categories: low, middle, and high. A self-administered short form of the International Physical Activity Questionnaire was used to evaluate physical activity status of subjects [35]. All anthropometric measurements were performed by a trained dietitian. Weight was measured to nearest 0.1 kg using a Seca scale (Seca, Germany), while the participant was barefoot and lightly dressed. A tape measure with a precision of 0.1 cm was used to measure height while the participant was in a standing position without shoes. Body mass index (BMI) of subjects was calculated as weight in kilograms divided by the square of height in meters. Waist circumference was measured at the narrowest part between the rib cage and above the umbilicus using a flexible unscratched tape to the nearest 0.1 cm over light clothing at the end of an exhalation. Systolic blood pressure (SBP) and diastolic blood pressure (DBP) of the participants were measured after 15 min rest in a sitting position. These measurements were performed using a standardized mercury sphygmomanometer, and the mean of the two measurements was record as the participant BP.

### Mental health and appetite assessment

Information on the mental health component was collected using a self-administered Depression, Anxiety and Stress Scale-21 Items (DASS-21) questionnaire. DASS-21 is a set of three subscales including depression, anxiety, and stress; each of these three subscales contains seven items. The validity and reliability of this questionnaire have been previously assessed and indicated good results [36, 37], such that Cronbach’s alphas for the DASS questionnaire in Iranian participants have been reported as follows: 0.77, 0.79, and 0.78 for depression, anxiety, and stress, respectively [36]. The instructions on how to complete this scale were described for all participants. The items are rated on a 4-point Likert scale, ranging from zero (“did not apply to me at all”) to 3 (“applied to me very much or most of the time”). The relevant item scores were summed and multiplied by 2 to give a total score for each subscale, which ranges from 0 to 42. After calculating the overall score, individuals were categorized into 5 categories: normal, mild, moderate, severe, and extremely severe based on cut-off scores proposed by Lovibond and Lovibond [38]. A higher score on every subscale represented a greater severity of mood disruption.

Appetite sensation was evaluated using the 100-mm Visual Analog Scale (VAS) questionnaire. The VAS questionnaire, with prior evidence of validity and reliability, includes seven items about feelings of hunger, satiation, fullness, prospective food consumption, and the desire to eat something sweet, salty, or fat [39]. Participants are requested to make a mark on each 100 mm line corresponding to their feeling. VAS scores are determined by measuring the distance from the left side of the line to the mark.

### Biochemical assessments

Fasting blood samples were obtained from all participants after 12-h overnight fasting. Plasma and serum were immediately separated by centrifugation at 4500 rpm for 10 min at 4 °C, and aliquots were frozen at – 80 °C until they were assayed. Serum glucose, triglyceride (TG), total cholesterol (TC), and high-density lipoprotein cholesterol (HDL-C) concentrations were measured enzymatically using commercial kits (Pars Azemoon, Tehran, Iran). Friedewald’s equation was used to calculate serum low-density lipoprotein cholesterol (LDL-C) from serum TC, TG, and HDL-C concentrations [40]. Plasma α-MSH and AgRP concentration were assayed using commercially available enzyme-linked immunosorbent assay kits (Bioassay Technology Laboratory, Shanghai Korean Biotech, Shanghai City, China) according to the manufacturer’s protocol. The minimum detectable concentrations of α-MSH and AgRP were 5.07 ng/L and 1.03 pg/ml, respectively. Similarly, ELISA kits were used to measure serum insulin level. Insulin resistance indices, including homeostasis model assessment-insulin resistance index (HOMA-IR) and quantitative insulin sensitivity check index (QUICKI), were calculated using the standard formula [41, 42]. Atherogenic index of plasma (AIP) was calculated as a logarithmic transformation of TG to HDL-C ratio [43].

### Genotyping

The extraction of genomic DNA from whole blood samples was performed using a standard phenol/chloroform technique. The participants were genotyped for the selected polymorphism (rs17782313) using the polymerase chain reaction-restricted length polymorphism (PCR–RFLP) technique. Template primers used for the PCR amplification of the rs17782313 were as follows: forward: 5′ AAGTTCTACCTACCATGTTCTTGG3′; reverse, 5′ TTCCCCCTGAAGCTTTTCTTGTCATTTTGAT 3′ (Macro-gene, Korea). The PCR amplification was performed with an initial denaturation at 95 °C for 2 min, denaturation at 95 °C for 30 s (35 cycles), annealing at 58 °C for 30 s, and 30 s of extension at 72 °C. An additional extension occurred at 72 °C for 5 min. Amplified DNA (7 μl) was digested with 0.5 μl of BclI restriction enzyme (10 U/μl, Fermentas, Germany) and 2 μl of 10× restriction G-buffer at 56 °C overnight. The digested PCR products were analyzed by electrophoresis on 2% agarose gel and stained with green viewer and then visualized on a Gel Doc-system (U.V.P. Company, Cambridge, UK). After electrophoresis, the T allele was distinguished as fragments with length of 30 and 107 bp and the C allele as a 137 bp fragment.

### Statistical analysis

The normality of data was tested by descriptive measures such as coefficients of skewness and kurtosis, mean, and standard deviation [44]. Continuous variables that were not normally distributed (glucose, TG, HOMA-IR, and insulin) were log transformed prior to analysis. According to previous studies [10, 14], participants were categorized by genotype groups. Descriptive analyses are expressed as means and standard deviations (Mean ± SD) for normally distributed continuous variables, the median (25th and 75th percentile) for those with a skewed distribution, and frequency (%) for categorical variables. Sex-stratified one-way analysis of variance (ANOVA) and chi-square tests were used to compare quantitative and qualitative variables, respectively, across the genotype groups. Sex-stratified multivariate multinomial logistic regression was used to examine the association between diet quality indices and rs17782313 genotypes, adjusting for confounding variables (age, physical activity, SES, and WC).

The interactions between rs17788313 and adherence to diet quality indices (DASH score and MDS) on cardio-metabolic risk factors were examined using an ANCOVA multivariate interaction model, after adjusting for covariates. All interaction analyses were conducted according to sex, and the homozygote group with the major allele was used as the reference group. Significant interactions were depicted as plots to visualize results and help their illustrations. Data were analyzed using SPSS 21(SPSS, Inc., Chicago, IL). A *p* value of ≤ 0.05 was considered statistically significant.

## Results

The frequencies of C and T alleles of rs17782313 were 37% and 63%, respectively. Genotype prevalence among study subjects was as follow: TT (44.7%), TC (36.2%), and CC (19.1%). The genotype distribution of MC4R rs17782313 was not in Hardy Weinberg equilibrium (*p* < 0.05). Table 1 shows sex-stratified general characteristics, clinical, and biochemical parameters of participants according to the MC4R rs17782313 genotypes. Distribution of SES for women was substantially different among genotypes of the MC4R rs17782313 (*p* = 0.024). Females with higher levels of SES were in the TT genotype group. On the other hand, females who placed in middle SES group had the CT genotype. Among women, those who had the lowest levels of SES were C allele carriers. No significant differences were observed regarding other demographic variables or mental health parameters across MC4R rs17782313 genotypes in either females or males. Apart from that, sex-stratified analysis for the association between biochemical parameters and MC4R rs17782313 genotypes showed that males with the homozygous minor allele genotype were more likely to have higher serum glucose concentrations (*p* = 0.040). Additionally, men in the CC genotype group showed lower plasma AgRP level compared with those in other genotype groups (CT, TT) (*p* = 0.025). Distribution of subjects regarding other clinical and biochemical parameters across MC4R rs17782313 genotypes was not significantly different in either male or female groups. Table 2 presents a sex-stratified analysis for the association between dietary indices and MC4R rs17782313 genotypes. There was no significant association between dietary quality indices (DASH and MDS) and MC4R rs17782313 genotypes in either crude or multivariate-adjusted models in males. However, among females, after adjusting for different potential confounders (age, physical activity, SES, and WC), an inverse association between the DASH score and the odds of having the CC genotype was found when compared to the TT genotype group (OR, 0.83; 95% CI, 0.70–0.99)**.**

**Table 1:** Comparison general characteristics, clinical, and laboratory parameters of participants according to MC4R rs17782313 genotypes

	Women				Men				
	TT	TC	CC	P*		TT	TC	CC	P*
**Age (year)**	38.46 (8.95)	39.07 (8.32)	36.31 (8.54)	0.628	39.21 (6.75)		37.26 (6.07)	40.44 (3.64)	0.527
**WC**	103.06 (10.43)	105.33 (9.31)	101.14 (7.14)	0.439	113.54 (5.89)		113.57 (7.45)	112.56 (5.20)	0.651
**BMI (kg/m**^**2**^**)**	35.02 (4.25)	35.27 (3.41)	34.99 (3.80)	0.990	33.90 (2.45)		33.55 (2.85)	33.64 (2.53)	0.622
**Physical activity level (%)**				0.129					0.778
Low	33.3	42.9	23.8		51.7		34.5	13.8	
Moderate	26.7	46.6	26.7		44.4		44.4	11.2	
High	66.7	20.0	13.3		54.6		22.7	22.7	
**Marital status (%)**				0.883					0.827
Married	36.7	36.7	22.6		49.1		35.6	15.3	
Single	40.0	40.0	20.0		60.0		20.0	20.0	
**SES (%)**									
Low	0	50.0	50.0	**0.024**	0		0	0	0.904
Middle	37.0	38.9	24.1		59.1		18.2	22.7	
High	57.2	35.7	7.1		46.8		40.4	12.8	
**Depressing (%)**				0.503					0.439
Normal	45.8	37.5	16.7		48.7		30.8	20.5	
Mild	18.2	54.5	27.3		45.5		54.5	0.0	
Moderate	33.3	23.8	42.9		53.8		30.8	15.4	
Severe	42.8	28.6	28.6		66.7		0.0	33.3	
Extremely severe	22.2	66.7	11.1		66.7		33.3	0.0	
**Anxiety (%)**				0.550					0.909
Normal	40.0	45.0	15.0		48.4		35.5	16.1	
Mild	50.0	37.5	12.5		40.0		40.0	20.0	
Moderate	33.3	38.9	27.8		61.1		33.3	5.6	
Severe	37.5	25.0	37.5		50.0		25.0	25.0	
Extremely severe	38.9	38.9	22.2		42.8		28.6	28.6	
**Stress (%)**				0.275					0.602
Normal	34.8	43.5	21.7		50.0		35.3	14.7	
Mild	30.8	30.8	38.4		41.7		33.3	25.0	
Moderate	42.9	38.1	19.0		50.0		35.7	14.3	
Severe	38.5	46.1	15.4		80.0		0.0	20.0	
Extremely severe	100.0	0.0	0.0		50.0		50.0	0.0	
**Appetite**	32.39 (9.49)	31.89 (6.80)	33.75 (7.62)	0.783	34.21 (10.75)		34.70 (10.00)	35.78 (7.93)	0.880
**LDL-C (mg/dl)**	126.9 (29.6)	118.56 (31.06)	108.91 (28.09)	0.161	121.48 (25.94)		118.30 (30.01)	117.58 (22.48)	0.471
**HDL (mg/dl)**	48.46 (10.27)	46.37 (10.19)	49.44 (6.46)	0.549	40.91 (6.79)		43.35 (9.22)	45.00 (10.46)	0.388
**Cholesterol (mg/dl)**	197.71 (33.88)	185.74 (35.45)	177.75 (28.62)	0.146	188.88 (28.64)		187.78 (31.20)	193.56 (28.58)	0.918
**TG (mg/dl)**	98.5 (77.75, 141.00)	93.00 (76.00, 125.00)	98.50 (73.25, 109.75)	0.638	120.00 (78.00, 168.50)		121.00 (90.00, 164.00)	123.00 (93.00, 176.50)	0.869
**AIP**	− 0.02 (0.24)	− 0.03 (0.23)	− .09 (0.17)	0.631	0.11 (0.26)		0.09 (0.25)	0.12 (0.29)	0.907
**Glucose (mg/dl)**	92.00 (85.25, 95.00)	91.00 (81.00, 101.00)	90.00 (85.25, 94.00)	0.834	93.00 (87.00, 101.50)		90.00 (83.00, 97.00)	101.00 (89.00, 159.50)	**0.040**
**Insulin, U/ml**	13.55 (9.28, 24.10)	19.60 (9.70, 27.30)	13.75 (9.00, 16.23)	0.306	11.40 (8.35, 17.70)		10.90 (8.40, 19.50)	11.00 (9.75, 25.80)	0.507
**HOMA-IR**	3.17 (2.00, 5.56)	4.82 (2.01, 6.58)	3.15 (2.09, 3.86)	0.409	2.70 (1.76, 4.06)		2.61 (1.87, 5.04)	4.16 (2.75, 6.99)	0.077
**QUICKI**	0.32 (0.03)	0.32 (0.04)	0.33 (0.03)	0.466	0.33 (0.03)		0.33 (0.03)	0.31 (0.03)	0.126
**SBP (mmHg)**	111.86 (14.48)	117.26 (16.85)	111.19 (14.40)	0.404	116.67 (11.43)		118.70 (15.83)	116.67 (14.14)	0.816
**DBP (mmHg)**	76.50 (10.11)	79.15 (14.55)	75.50 (11.68)	0.644	76.51 (10.86)		78.26 (10.93)	75.56 (12.10)	0.584
**α-MSH (ng/l)**	2.16 (2.12–2.20)	2.26 (2.17–2.36)	2.26 (2.11–2.41)	0.153	2.29 (2.20–2.37)		2.39 (2.25–2.52)	2.18 (2.09–2.27)	0.105
**AgRP (pg/ml)**	1.33 (1.29–1.37)	1.44 (1.36–1.51)	1.41 (1.29–1.53)	0.082	1.49 (1.42–1.57)		1.57 (1.46–1.68)	1.33 (1.25–1.41)	**0.025**

Data are presented as mean (SD) or median (25 and 75 percentiles)

*BMI* body mass index, *WC* waist circumference, *SES* socio-economic status, *HOMA-IR* homeostasis model assessment of insulin resistance, *LDL-C* low density lipoprotein cholesterol, *HDL* high-density lipoprotein, *SBP* systolic blood pressure, *DBP* diastolic blood pressure, *TG* triglyceride, *QUICKI* quantitative insulin sensitivity check index, *AgRP* agouti-related protein, *α-MSH* alpha melanocyte stimulating hormone, *AIP* atherogenic index of plasma

*Analysis of variance for continuous variables and *χ*^2^ test for categorical variables

**Table 2:** Odd’s ratio (OR) and confidence interval (CI) for the association between dietary quality indices and MC4R rs17782313 genotypes

	Women			Men		
	TT	TC	CC	TT	TC	CC
**MDS (total score)**						
**Crude**	1 (Ref.)	1.04 (0.75–1.44)	1.10 (0.75–1.62)	1 (Ref.)	1.40 (0.89–2.18)	1.68 (0.94–2.95)
Model 1*	1 (Ref.)	1.08 (0.77–1.52)	1.12 (0.73–1.72)	1 (Ref.)	1.50 (0.94–2.40)	1.66 (0.92–3.02)
Model 2**	1 (Ref.)	1.08 (0.77–1.52)	1.11 (0.71–1.71)	1 (Ref.)	1.48 (0.92–2.37)	1.76 (0.96–3.22)
**DASH score (total score)**						
**Crude**	1 (Ref.)	0.94 (0.83–1.06)	0.87 (0.75–1.01)	1 (Ref.)	1.09 (0.95–1.24)	1.13 (0.95–1.35)
**Model 1**	1 (Ref.)	0.94 (0.82–1.07)	0.86 (0.73–1.01)	1 (Ref.)	1.16 (0.99–1.35)	1.19 (0.97–1.46)
**Model 2**	1 (Ref.)	0.94 (0.82–1.06)	**0.83 (0.70**–**0.99)**	1 (Ref.)	1.17 (1.00–1.36)	1.18 (0.95–1.45)

The multivariate multinomial logistic regression was used for estimation of ORs and confidence interval (CI)

*Adjusted for age, physical activity, and socio-economic status

**Additionally adjusted for waist circumference. Indicates statistically significant values as *p* < 0.05

**Fig. 1:**
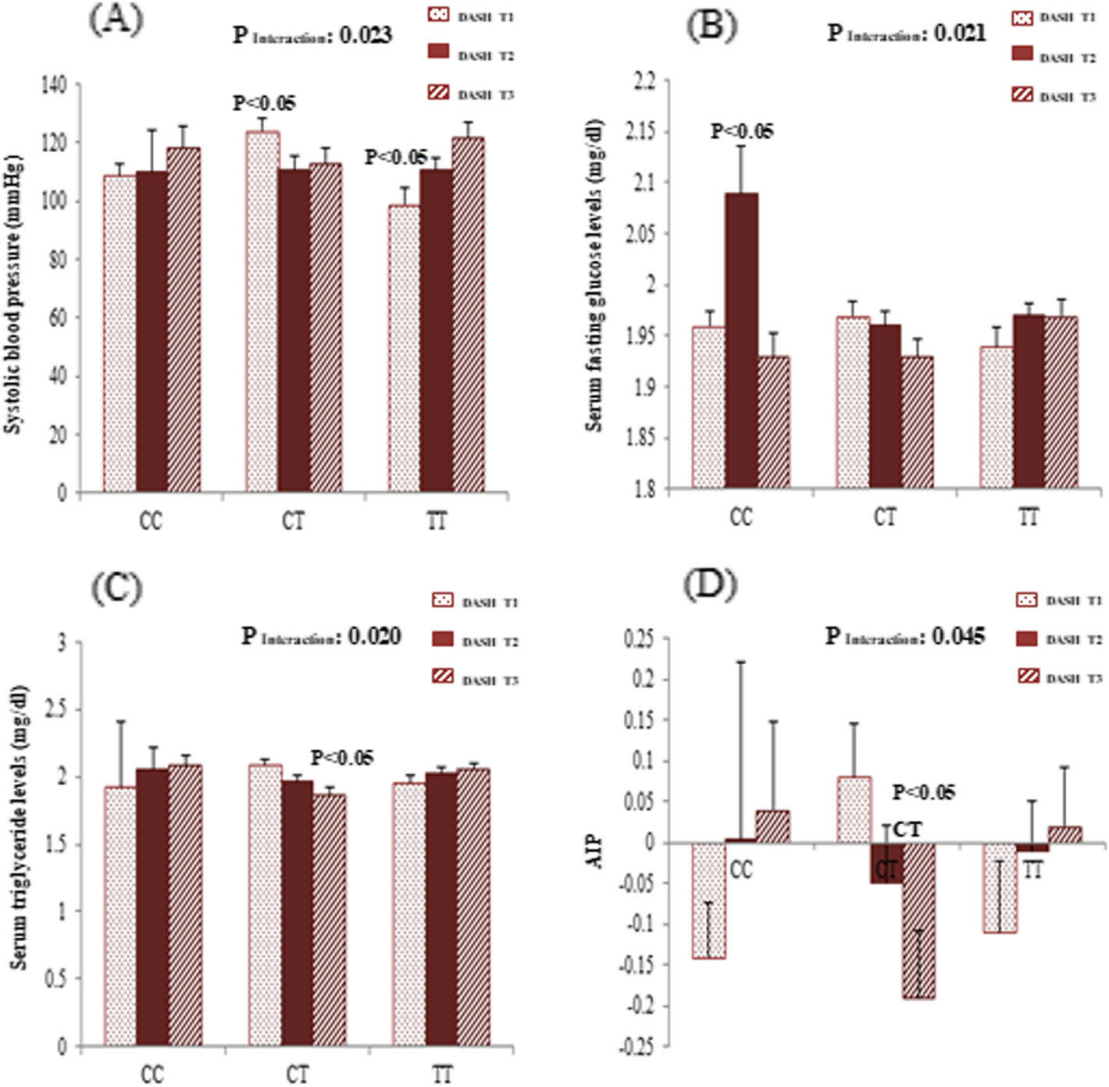
Interaction between MC4R rs17782313 and DASH score on systolic blood pressure (**a**), serum concentration of glucose (**b**), serum concentration of triglyceride (**c**), and atherogenic index of plasma (AIP) (**d**) among women. The bars indicate mean. Error bars: SE of means

**Fig. 2.**
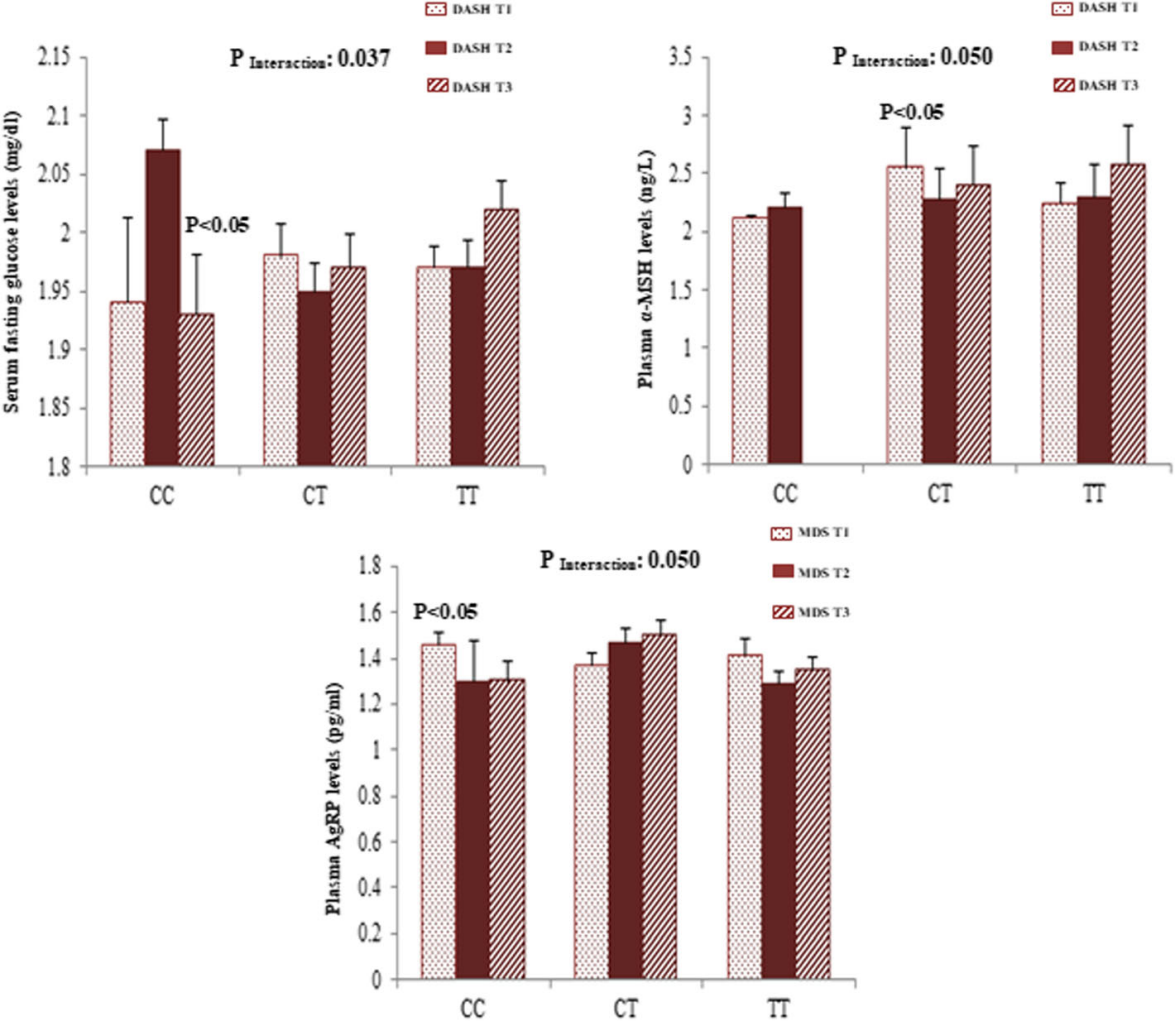
Interaction between MC4R rs17782313 and DASH score on serum concentration of glucose (**a**) and plasma concentration of α-MSH (**b**) among men. Interaction between MC4R rs17782313 and MDS on plasma concentration of AgRP (**c**) among women. The bars indicate mean. Error bars: SE of means. *p* values of interactions were adjusted for age, WC, physical activity, and socio-economic status

Gene-diet interaction analyses were performed in order to assess the potential interaction between MC4R rs17782313 polymorphism and adherence to DASH score and MDS on cardio-metabolic risk factors and hypothalamic hormones. Significant sex-stratified interactions are depicted in Figs. 1 and 2. From these analyses, significant interactions were found between adherence to DASH score and MC4R rs17782313 genotypes on SBP (*p*_Interaction_ = 0.023), AIP (*p*_Interaction_ = 0.045), serum glucose (*p*_Interaction_ = 0.021), and TG (*p*_Interaction_ = 0.020) concentrations among females, where females who were assigned to the second tertile of DASH had significantly lower SBP levels (*p* < 0.05) compared with those in the first tertile if they were heterozygous for the MC4R rs17782313. In contrast, females with a homozygous major allele genotype had lower means of SBP when they had the lowest adherence to DASH (*p* < 0.05). On the other hand, higher means of glucose concentration were seen in homozygous females for the minor allele who were assigned to the second tertile of DASH (*p* < 0.05). Additionally, females with the highest adherence to DASH score who carried the CT genotype had lower serum TG than those in the lowest DASH category (*p* < 0.05). Likewise, in CT-genotype carriers, the AIP mean was lower in females with the highest adherence to DASH than those in lowest category (*p* < 0.05). However, all of these significant interactions disappeared after adjustment for potential covariates. After adjustment for confounding variables, a significant interaction was revealed between MC4R re17782313 and DASH on plasma AgRP level among females (*p*_Interaction_ = 0.050) and on serum glucose concentration among males (*p*_Interaction_ = 0.037). In homozygous carriers of the risk allele, the plasma level of AgRP in females with the lowest adherence to DASH was significantly higher than those with the highest adherence (*p* < 0.05). Among males, CC-genotype carriers who were assigned to the highest tertile of DASH had the lowest serum glucose levels than males in other categories (*p* < 0.05). Additionally, in males, a significant interaction was observed between adherence to the MDS and this polymorphism in association with plasma α–MSH in the multivariate-adjusted interaction models (*p*_Interaction_ = 0.050). In particular, in CT genotype carriers, mean plasma α-MSH levels were higher in males with the lowest adherence to MDS, in comparison with those in second tertile (*p* < 0.05).

## Discussion

These results showed consistently significant interactions between adherence to diet quality indices and genetic predisposition of near variant MC4R (rs17782313) in relation to cardio-metabolic risk factors and hypothalamic hormones. We documented significant interactions between adherence to DASH score and MC4R rs17782313 genotypes in changing SBP, AIP, serum glucose, and TG among females. However, adjustment for different potential confounders eliminated these significant interactions. Another main finding in the present research was a significant interaction between MC4R rs17782313 polymorphism and DASH score on plasma AgRP level in females even after adjusting for confounding variables. Among males, there was evidence of gene-DASH score and gene-MDS interactions in changing serum glucose concentration and plasma α-MSH level in multi-adjusted interaction models. Moreover, interestingly, an inverse and strong association was revealed between adherence to DASH score and the chance of having the CC genotype in a multivariate-adjusted model among females. Additionally, males with the homozygous minor allele genotype had higher serum glucose and lower plasma AgRP levels than those in the other genotype groups. The minor allele frequency of MC4R rs17782313 in our research (37%) was slightly higher than the range of reported values in other population published studies (14–28%) according to the HapMap database. These discrepancies in reported frequencies may be due to differences in dietary habits, lifestyle, and other demographic characteristics among various ethnic groups.

In the current study, generally, the association between the MC4R rs17782313 polymorphism and some cardio-metabolic traits depends on the degree of adherence to healthy dietary patterns as assessed based on DASH. Female homozygous carriers of the risk allele presented higher means of glucose concentration when placed in the second tertile of DASH. Among females, CT-genotype carriers with a good adherence to DASH showed a better metabolic profile (lower TG, SBP, and AIP). Likewise, a similar result was observed for glucose levels in males. These sex-based differences for the effect of rs17782313 in obesity-related traits are recognized with previous studies [45]. Although the underlying mechanisms for these gender-related differences remain poorly understood, differences in hormonal status and regional deposits of adipose tissue may contribute to these discrepant outcomes [46].

These results provide evidence that higher adherence to the DASH diet may protect carriers of the risk allele against elevation in some cardio-metabolic risk factors. Despite the fact that the specific interaction of rs17782313 variant with dietary quality indices on cardio-metabolic risk factors is still not known, our findings are in line with experimental studies in which MC4R knockout mice exhibited hyperphagia and weight gain when they were fed a high fat diet [47]. A few studies have assessed the interaction between MC4R SNPs and dietary intakes on obesity-related traits like metabolic syndrome [48] with inconclusive results [28]. For instance, a study of the gene-diet interaction by Azorin et al. revealed that carriers of the variant alleles for the MC4R rs17782313 had higher type 2 diabetes risk when adherence to the Mediterranean dietary pattern was low [17]. Similar interactions have also been reported by two prospective cohorts of American women and men [49]. They found that improving adherence to healthy dietary patterns as assessed by the Alternate Healthy Eating Index 2010 (AHEI-2010) and Dietary Approach to Stop Hypertension (DASH) could attenuate the genetic association with long-term weight gain predominantly in subjects with a genetic predisposition to obesity. Even though the exact underlying mechanisms of these observed interactions are still not clear, it seems that the favorable modulating effects of these indices on the association of the rs17782313 genotypes with cardio-metabolic risk factors can be attributed to desirable food groups such as vegetables, fruits, whole grains, seafood and plant proteins, nuts and more emphasis on polyunsaturated or monounsaturated to saturated fat ratio [50]. Considerable evidence has shown that adherence to these quality indices is inversely related to chronic disease [22]. Thus, it is not surprising that low compliance with these diet quality indices can promote effects of greater genetic predisposition to cardio-metabolic risk factors in C allele carriers of rs17782313. In the present study, homozygote males with the minor allele had higher means of serum glucose levels compared with other genotypes (TT and TC). These findings were in agreement with evidence from earlier studies in which the rs17782313 variant, especially mutant homozygote, was significantly related to some cardio-metabolic factors such as hypertriglyceridemia [14], low HDL-cholesterol, high LDL-cholesterol and total cholesterol [51], and insulin resistance indices [52]. Similarly, several observational studies reported a higher risk of type 2 diabetes [53, 54] and metabolic syndrome [55] in carriers of the minor allele of rs17782313. However, there were inconsistencies in results of these studies regarding the association between MC4R rs17782313 and metabolic traits [56]. Although the reason for these discrepant results is unclear, differences in study design and population characteristics such as ethnicity, age, and environmental factors may explain part of the inconsistency with these outcomes.

Another main and novel finding in the current study was the significant interaction between MC4R rs17782313 and diet quality indices on plasma hypothalamic hormones levels regardless of adjustment for potential confounders. Among the CC genotype group, females with the lowest adherence to DASH had higher adjusted means of plasma AgRP level. In addition, CT heterozygote male carriers who were assigned to the first tertile of MDS had higher adjusted means of plasma α-MSH concentration. On the other hand, male subjects with the CC genotype had lower means of plasma AgRP levels than other genotype groups (CT, TT). It seems that minor C allele carriers of rs17782313 (CT or CC), compared with the wild-type genotype (TT), may be more predisposed to high plasma levels of hypothalamic hormones when the adherence to healthy dietary patterns is low. As far as we know, there is no human study on the relationship between α-MSH, AgRP, and MC4R polymorphisms which makes it difficult to compare our findings. However, many reports have indicated that obese adults have elevated plasma levels of both α-MSH and AgRP [57, 58]. Despite the fact that the function of α-MSH and AgRP in circulation, especially in relation to energy balance, is still not elucidated, it seems that obesity-induced hyperleptinemia—by making hypothalamic resistance to function of these hormones and deregulation in hypothalamic expression of these neuropeptides—may contribute to positive energy balance and development of adiposity [59, 60].

Noticeably, in the present study, an inverse association between adherence to the DASH diet and the likelihood of having the CC genotype was detected. Our results were in accordance with previous observations in which the variant homozygote was related to higher intakes of total energy and dietary fat [54]. A recent study in this regard by Khaliltehrani et al. [10] has similarly noted that this variant is related to a variety of energy and macronutrient intakes. Nevertheless, existing literature regarding the association between dietary intake and rs17782313 is controversial [61]. Despite the fact that the mechanisms behind the effect of MC4R rs17782313 on obesity and its related metabolic phenotypes are less understood, there is a suggestion that this variant may play an important role in central appetite control and eating behaviors [62].

The current study has several clear limitations that should be taken to account when interpreting the results. First, the cross-sectional nature of the study makes it impossible to extract causal inference. Nevertheless, these findings can be used to generate hypotheses for prospective cohort studies. Second, the relatively small sample size may decrease the statistical power. Thus, our findings should be taken with caution and need to be replicated in larger longitudinal studies. Third, since obese participants tend to underreport their dietary intakes, potential biases caused by under-reporting may lead to null results [63]. However, subjects with extreme dietary intake values were excluded from the analysis to minimize the influence of this measurement error. Additionally, although we tried to control major confounders, residual confounding due to unmeasured confounders may exist. Fourth, it is known that multiple gene variants may contribute to pathogenesis of obesity and related metabolic phenotypes, but only one single nucleotide polymorphism from a single gene was assessed in our study. Finally, because this study was performed in Tabriz, a major city in the northwest of Iran with different dietary intake and other lifestyle factors, our results may not be extrapolated to all Iranians.

The main strength of the present study is that, according to our knowledge, it is the first study to evaluate the interaction between MC4R rs17782313 and diet quality indices on cardio-metabolic traits and hypothalamic hormones. Indeed, a better understanding of these gene-diet interactions may provide the best strategy for personalized nutritional advice tailored to the patient’s genotype in the management of obesity and its related consequences. An additional strength was using a reliable [29] and validated [30] FFQ to assess dietary intake.

In summary, the present study described, for the first time, a statistically significant gene-diet interaction of the MC4R rs17782313 with adherence to healthy dietary patterns assessed according to DASH and MDS on cardio-metabolic traits and hypothalamic hormones in both sexes. When adherence to DASH and MDS was low, the obesity risk allele was associated with higher means of AgRP and α–MSH among women and men, respectively. Overall, our findings suggest that low compliance with these diet quality indices can promote effects of greater genetic susceptibility to cardio-metabolic risk factors in C allele carriers of rs17782313. Consequently, replication in prospective cohort studies and among different populations is recommended to confirm this finding.

## Data Availability

All of the data are available with reasonable request from the corresponding author.
